# *Plasmodium berghei* Gamete Egress Protein is required for fertility of both genders

**DOI:** 10.1002/mbo3.1038

**Published:** 2020-04-30

**Authors:** Maria Andreadaki, Tomasino Pace, Felicia Grasso, Inga Siden‐Kiamos, Stefania Mochi, Leonardo Picci, Lucia Bertuccini, Marta Ponzi, Chiara Currà

**Affiliations:** ^1^ FORTH Institute of Molecular Biology and Biotechnology Heraklion Greece; ^2^ Dipartimento di Malattie Infettive Istituto Superiore di Sanità Roma Italy; ^3^ Core Facilities Istituto Superiore di Sanità Rome Italy

**Keywords:** Anopheles, gametocyte egress, malaria, osmiophilic bodies

## Abstract

Male and female *Plasmodium* gametocytes ingested by the *Anopheles* mosquitoes during a blood meal egress from the red blood cells by rupturing the two surrounding membranes, the parasitophorous vacuole and the red blood cell membranes. Proteins of the so‐called osmiophilic bodies, (OBs), secretory organelles resident in the cytoplasm, are important players in this process. Once gametes emerge, the female is ready to be fertilized while the male develops into motile flagellar gametes. Here, we describe the function(s) of PBANKA_1115200, which we named Gamete Egress Protein (GEP), a protein specific to malaria parasites. GEP is restricted to gametocytes, expressed in gametocytes of both genders and partly localizes to the OBs. A mutant lacking the protein shows aberrant rupture of the two surrounding membranes, while OBs discharge is delayed but not aborted. Moreover, we identified a second function of GEP during exflagellation since the axonemes of the male flagellar gametes were not motile. Genetic crossing experiments reveal that both genders are unable to establish infections in mosquitoes and thus the lack of GEP leads to a complete block in *Plasmodium* transmission from mice to mosquitoes. The combination of our results reveals essential and pleiotropic functions of GEP in *Plasmodium* gametogenesis.

## Graphical abstract

PBANKA_1115200, which we named Gamete Egress Protein (GEP), is a gametocyte protein found only in malaria parasites. GEP partly localizes to osmophilic bodies. A mutant lacking this protein showed aberrant rupture of the parasitophorous vacuole and the red blood cell membranes. Axonemes of the male flagellar gametes *gep(‐)* were not motile.

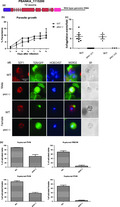

## 1 INTRODUCTION

The sexual stage of *Plasmodium* takes place after the uptake of gamete precursors, the female and male gametocytes, by the mosquito vector during an infected blood meal. In a few minutes, gametocytes develop into gametes of the two genders that fuse to form a zygote. In 18–20 hr, it develops into an elongated motile ookinete that passes through the midgut epithelium and forms a sporogonic oocyst. During the next two weeks, approximately, oocysts rupture releasing infectious sporozoites that travel to the salivary glands from where, in turn, they are injected into a new host.

Gametogenesis entails a complex developmental process, triggered by a change in temperature and by the mosquito factor xanthurenic acid (Billker et al., 1998). These changes activate a cGMP‐dependent signaling pathway resulting in Ca^2+^ mobilization from internal stores and activation of the calcium‐dependent protein kinase 4 (CDPK4). This master regulator is essential for male gamete maturation, which entails three mitotic divisions, and assembly of axonemes to form eight motile gametes (Billker et al., 2004). Blood stage gametocytes develop within a parasite‐specific compartment, the so‐called parasitophorous vacuole (PV). The egress of activated male and female gametocytes from the host RBC involves the sequential rupture of the PV membrane (PVM) and the RBC membrane (RBCM) (Deligianni et al., 2013; Sologub et al., 2011). Nearly concomitant with the RBCM rupture, the male gamete flagella start beating detaching from the residual body of the cytoplasm (Andreadaki et al., 2018). Female gametogenesis is regulated by translational repression of messenger RNAs; mRNA turnover influences gene expression in a stage‐specific manner. This was shown for the DDX6‐class RNA helicase, DOZI (development of zygote inhibited), usually found in a complex with mRNA species in cytoplasmic bodies of females (Mair et al., 2006). Gene deletion of *DOZI* led to inhibition of the ribonucleoprotein complexes formation with subsequent degradation of at least 370 transcripts. However, female gametogenesis morphologically comprises, as far as is known, only the egress from the host RBC.

Proteins localized in specialized secretory organelles called osmiophilic bodies (OBs) have been identified as having specific roles in the egress process. In the rodent *Plasmodium berghei,* mutants lacking *Plasmodium* male development gene – 1 (MDV1) (Ponzi et al., 2009) and gamete egress and sporozoite traversal protein (GEST) (Talman et al., 2011), detected in OBs of both genders, are unable to rupture the PVM, while lack of G377, a female‐specific OB factor, cause a slight delay in the egress of female gametes (Olivieri et al., 2015). MTRAP, a member of the thrombospondin‐related anonymous protein (TRAP) family, is also required for PVM rupture (Bargieri et al., 2016). The putative pantothenate transporter PAT, a membrane component of the OBs, has a function in the release of OB contents, and gametes lacking *pat* remain trapped inside the PVM (Kehrer, Frischknecht, & Mair, 2016). A *P. berghei* Ferlin‐like protein (FLP) localizes to vesicles distinct from OBs and is also involved in gamete egress (Obrova, Cyrklaff, Frank, Mair, & Mueller, 2019). Gametes lacking FLP remain trapped inside PVM and RBC membranes. A male‐specific perforin‐like protein, PPLP2, is specifically required for RBCM rupture (Deligianni et al., 2013). Other studies have shown that male gametogenesis is dependent upon the APC/C (Anaphase Promoting Complex/Cyclosome) protein for chromosome condensation and cytokinesis (Wall et al., 2018); in its absence, cytokinesis is abnormal and no gametes are formed, resulting in a similar phenotype to mutants lacking the CDC20 homolog (Guttery et al., 2012). The aspartic protease plasmepsin X (PMX) is also involved in *P. berghei* gametocyte egress. Inhibition of PMX activity through treatment with the aspartic protease inhibitor 49c causes a 10‐fold decrease exflagellation rate and prevents rupture of the RBCM in both female and male gametocytes (Pino et al., 2017).

Flagellar proteins are also required for proper axoneme formation. PF16 functions in maintaining microtubule structure, and in its absence, flagellar movement is abnormal and fertility is reduced (Straschil et al., 2010). In parasites lacking the SAS‐6 protein, a component of the basal body and centriole, flagellar motility, and nuclear allocation are affected leading to reduced fertility (Marques et al., 2015). A mutant lacking Pbmap‐2 (Tewari, Dorin, Moon, Doerig, & Billker, 2005) fails to release flagellated gametes, while the serine/arginine‐rich (SR) protein kinase (SRPK) is essential for male gamete formation (Tewari et al., 2010). Furthermore, the stage‐specific actin II is required both for axoneme activation and egress of male gametocytes (Deligianni et al., 2011). A role in axoneme assembly and flagellum formation during male gamete development is played by kinesin‐8B (Zeeshan et al., 2019).

In this work, we characterized a *P. berghei* (PBANKA_1115200), here named Gamete Egress Protein (GEP) due to its essential role(s) in gametogenesis of both male and female gametocytes. GEP is expressed in sexual blood stages and partly localizes to the OBs. When GEP is lacking, gametocytes develop normally but their maturation into gametes is severely affected. In males, axonemes are formed though they do not beat. Egress from the host RBC is also delayed in both genders and transmission to the mosquito is completely abrogated. Overall, our results suggest an important and pleiotropic function of GEP during *Plasmodium* transmission to the vector.

## 2 MATERIAL AND METHODS

### 2.1 Mosquito and parasite strains

*Anopheles gambiae* mosquitoes were reared as described in Facchinelli et al., 2015. *Plasmodium berghei* ANKA 8417HP strain was used throughout the study (Janse et al., 1989) and was maintained in CD1 mice. Transfections were performed as described in Janse, Franke‐Fayard, and Waters (2006). Mosquito infections were carried out by offering *Anopheles gambiae* strain G3 to mice infected with WT and mutant parasites. Mice were matched to have a parasitemia of roughly 5%–10% and a similar exflagellation rate in each experiment. Mosquito midguts were dissected after 13 days.

Synchronous infections were established by intravenous injection of purified schizonts (Janse & Waters, 1995). Blood was collected by heart puncture under anesthesia, and leukocytes were removed using Plasmodipur leukocyte filters (Euro Diagnostica). Schizont or gametocyte infected erythrocytes were purified through Nycodenz density gradient centrifugation (Janse & Waters, 1995).

### 2.2 The plasmid used for transfection and generation of knockout lines

The pBAT plasmid (Kooji, Rauch, & Matuschewski, 2012) was used to prepare the final transfection construct for the generation of *gep(‐)* parasites.

The transgenic parasite line was generated by introducing a 728 bp PCR fragment amplified from the 5’UTR of PBANKA_1115200 (primers L‐f and L‐r) in the restriction sites SacII‐EcoRI upstream to the mCherry coding region. A PCR fragment of 692 bp (primers R‐f and R‐r, Table A1) amplified in the 3’UTR of PBANKA_1115200 was inserted in restriction sites XhoI‐KpnI of the pBAT (Table A1). These two regions provided the targets used for double crossover recombination in the genomic locus. An hDHFR‐yFcu cassette, which encodes antifolate resistance, allowed for pyrimethamine selection of transfected parasites. Transfection and selection of transformed parasites were performed using standard genetic modification technologies for *P*. *berghei* (Janse, Franke‐Fayard, Mair, et al., 2006) using *P*. *berghei* HP as the parent parasite line.

After transfection of *P. berghei* 847HP as parental line, 5 × 10^5^ GFP‐fluorescent red blood cells were sorted and intravenously injected into two CD1 mice and kept under pyrimethamine selection. When parasitemia was established, sorting of fluorescent parasite and reinfection under selection was repeated two more times.

### 2.3 Production of GEP serum and antibodies dilutions

Bacterially expressed recombinant protein was produced using the pGEX‐6P‐1 vector into which a 453 bp PCR fragment, corresponding to a region within the 6th exon of PBANKA_1115200, coding for amino acids 284–399, was inserted. This fragment was amplified using the primers: REL‐10‐for and REL‐10‐rev (Table A1). This recombinant protein was used to produce a specific immune serum in BALB/c mice. About 50 µg of the recombinant protein and complete Freund's adjuvant were mixed to form a stable emulsion and injected intraperitoneally in the stomach area. The immunization procedure was repeated using 25 mg of antigen in incomplete Freund's adjuvant 28, 42, 56, and 70 days after the first injection. At day 84, the immunized mice were bled to obtain immune serum. Before the immunization cycle, blood (100 µl) was collected from the submandibular vein of each mouse to obtain preimmune serum.

Working dilutions of antibodies used in this study were α‐Pb377 mouse polyclonal, 1:300 in immunofluorescence assay (IFA), 1:2,000 in Western blot; α‐MDV1 mouse polyclonal, 1:400 in IFA, 1:2,000 in Western blot; α‐SET rabbit polyclonal (Pace et al.,^2006^) 1:200 in IFA; Alexa Fluor 488 α‐mouse TER‐119 (BioLegend) 1:200 in IFA.

### 2.4 Exflagellation assay

Gametocytes were activated after diluting infected blood 1:10 in activation medium (RPMI1640 with l‐glutamine, 25 mM HEPES, 2 g/L NaHCO3, 10% FBS, 50 μM XA, and pH 8.0) and incubating the samples for 15 min at 19°C.

### 2.5 Immunofluorescence assay

The TER‐119 antibody was used to label the outer leaflet of the RBCM, and anti‐SEP1 antibody was used as a marker for PVM. Gametocytes were activated, and samples were fixed in 4% formaldehyde at different time points after the activation. All steps were carried out at room temperature. For immunolabeling of WT and *gep*(‐) parasites with anti‐SEP1 antibody activated for 15 min, samples were permeabilized with 0.1% saponin for 30 s. The TER‐119 antibody was added diluted 1:200 in PBS with 2% normal goat serum for 1 hr incubation. Hoechst 33342 was used to stain DNA. Samples are washed twice with 1 × PBS before mounting in Vectashield (Vector laboratories).

### 2.6 Electron microscopy

Nycodenz‐purified gametocytes from synchronous infection were fixed in 2.5% glutaraldehyde, 2% paraformaldehyde, 2 mM CaCl_2_ in 0.1 M sodium cacodylate buffer, pH 7.4, overnight at 4°C and processed according to Perry and Gilbert (1979). Parasites were washed in cacodylate buffer and postfixed with 1% OsO4 in 0.1 M sodium cacodylate buffer for 1 hr at RT, treated with 1% tannic acid in 0.05 M cacodylate buffer for 30 min, and rinsed in 1% sodium sulfate in 0.05 M cacodylate buffer for 10 min. Postfixed specimens were washed, dehydrated through a graded series of ethanol solutions (30%–100% ethanol), and embedded in Agar 100 (Agar Scientific Ltd). Ultrathin sections, obtained by a UC6 ultramicrotome (Leica), were stained with uranyl acetate and Reynolds’ lead citrate and examined by an FEI/Philips EM 208S transmission electron microscope equipped with acquisition system/MegaView SIS camera at 80 kV.

### 2.7 Western blot analysis

Western blot analysis was performed using MINI TRANS‐BLOT® BioRad apparatus at constant voltage (100 V) for 1 hr, in transfer buffer (20% methanol, Tris 0.025 M, Glycine 0.192 M) onto Protran 0.22 µm membrane (Whatman). Incubation with antibodies was done. Primary and horseradish peroxidase‐conjugated secondary antibodies were incubated 1 hr in PBS‐Tween (0.05%), and the membrane was developed using the ECL system (SuperSignal West Pico, Thermo Scientific) according to the manufacturer's instructions.

## 3 RESULTS

### 3.1 GEP is conserved within the *Plasmodium* genus

**Figure 1:**
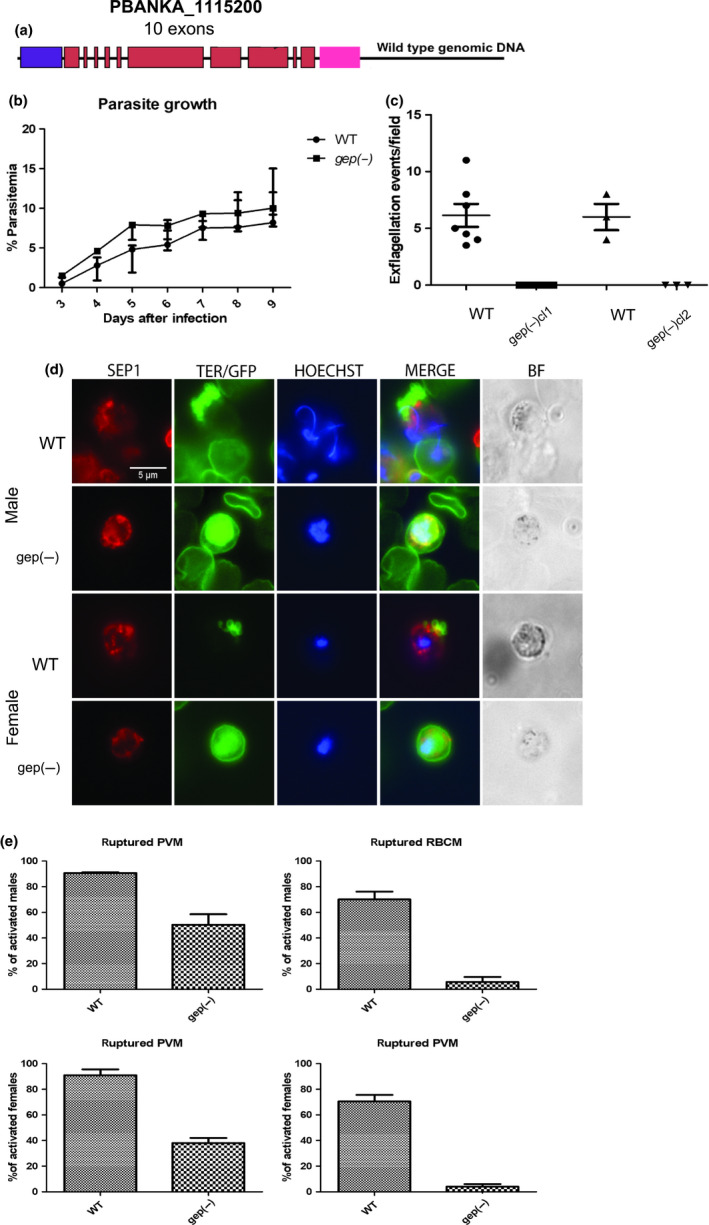
(a) Scheme of the *gep* locus (PBANKA_1115200) showing the coding region of 1,138 amino acids in 10 exons (red boxes). Violet and pink indicated, respectively, the 5’ upstream and the 3’ downstream regulatory regions. (b) Growth rate of *gep(‐)* parasites compared with WT parasites. Three independent experiments were done. No significant difference was detected (*t* test, *p*‐value = .4024). (c) The number of exflagellation events per field in the mutant line compared with WT. No exflagellation was detected in the mutant in 13 independent experiments. (d) Immunofluorescence assay on WT and *gep(‐*) male and female gametocytes activated for 15–20 min. Anti‐SEP1 antibodies were used to label the PVM (red) while TER‐119 (green) was used as RBCM marker. Mutant parasites constitutively express cytoplasmic GFP. Scale bar is 5 μm. (e) Percentage of activated males (upper panel) and females (lower panel) gametocytes in the WT and the *gep(‐)* cl1 with ruptured PVM or PVM and RBCM. Differences between WT and mutant are all statistically significant (Student's *t* test: male ruptured PVM: *p*‐value = .0074; female ruptured PVM: *p*‐value = .0041 male ruptured RBCM: *p*‐value = .0019; female ruptured RBCM: *p*‐value = .0022).

**Figure A1:**
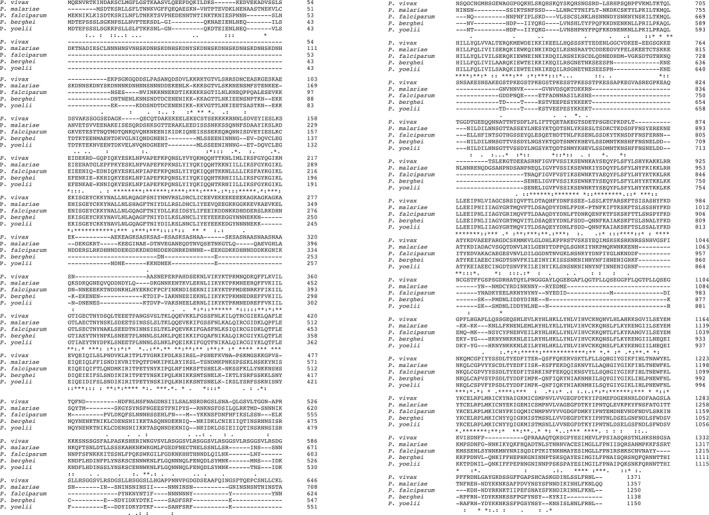
Alignment of GEP in *Plasmodium ssp P. berghei, P. falciparum, P. yoelii P. malariae, and P. vivax.* GEP is highly conserved through the main *Plasmodium* species affecting humans. The sequences are *P. falciparum* (PF3D7_0515600), *P. berghei* (PBANKA_1115200), *Plasmodium vivax* (PVP01_1018300), *Plasmodium malariae* (PmUG01_10029000), and *P. yoelii* (PY17X_02013). The sequences were obtained from the PLASMODB database (Aurrecoechea et al., 2009).

Gamete Egress Protein is a protein of 1,138 amino acids encoded by a 4,426 bp gene on chromosome 11 having 10 exons residing in a syntenic region (Figure 1a). The protein is well conserved within the *Plasmodium* genus as revealed by multiple sequence alignment comparing five different *Plasmodium* species (Figure A1). BLAST analysis of the protein did not retrieve any similar protein outside the genus or known protein domains or motifs. GEP has previously been detected in the proteome of both male and female gametocytes (Khan et al., 2005) and was shown to be phosphorylated in gametocytes (Garcia et al., 2018; Invergo et al., 2017). Interestingly, GEP was also identified in a proteomic list of proteins released during gametogenesis (Ponzi, unpublished) suggesting a specific function of this protein at this stage.

High throughput screening gene deletion analysis revealed that the protein was dispensable for growth of the asexual blood stages (Bushell et al., 2017).

### 3.2 A *gep* knockout mutant is strongly impaired in gametocyte egress from the red blood cell

**Figure A2:**
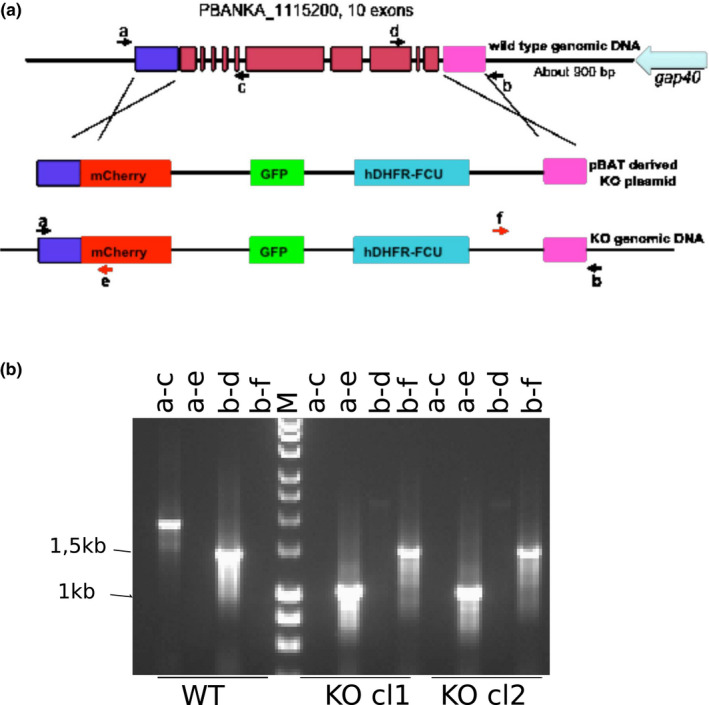
(a) Schematic representation of the strategy used to generate *gep(‐)* parasites. The 3’UTR region selected for plasmid design is about 900 bp upstream the adjacent *gap40* stop codon. (b) PCR genotyping of *gep(‐)* parasites clones 1 and 2. Transfected parasites were cloned to remove genomic WT contamination and the cloning verified by PCR. The primers are shown schematically in Figure 1a, and their sequences are found in Table A2. In neither *gep(‐)* clone WT contamination was detected. Expected sizes in diagnostic PCRs: left side of the gene, primers a‐c for WT, 1,866 bp and primers a–e for KO, 1,029 bp; right side: primers b–d for WT, 1,428 bp and primers b–f for KO, 1,524 bp.

To analyze the function of GEP, we generated a parasite line deficient in expression of the protein by gene disruption. The construct used for replacement of the *gep* coding region contained the hDHFR selection cassette flanked by 5′‐ and 3′‐UTR *gep* regions necessary to target the construct by double crossover integration into the chromosomal locus by homologous recombination. The 3’UTR region was selected about 900 bp upstream the stop codon of the adjacent gene *gap40* (Figure A2). A constitutively expressed GFP cassette contained in the plasmid enables the identification of transgenic parasites. Two independent transfection experiments were performed, and two transgenic parasite clones were obtained, *gep(‐)*cl1 and cl2 (Figure A2). Correct integration of the construct was confirmed by diagnostic PCR (Figure A2).

Growth of asexual blood stages was not impaired in either cloned lines lacking GEP compared with the WT (Figure 1b). This is consistent with previously reported results (Bushell et al., 2017). In the two independent *gep(‐)* clones, both gametocytemia and morphology of male and female gametocytes were also roughly similar to WT as scored in Giemsa stained blood smears from infected mice (Table A2).

Next, we looked at the release of motile male gametes (exflagellation) in vitro and their adherence to RBCs forming the so‐called exflagellation centers. Exflagellations from the WT and the two clonal *gep(‐)* lines were inspected under the light microscope 15–20 min after activation. In a total of 13 experiments, we did not detect a single exflagellation event in the mutant parasites (Figure 1c).

We then questioned whether the lack of GEP may affect gametocyte egress from the host cell upon activation. To answer this question, we analyzed the breakdown of both PVM and RBCM by immunolabeling gametocytes activated 15–20 min with antibodies against the SEP1 and TER‐119, which labels the PVM and the RBCM, respectively. Activated males can be distinguished from females by their enlarged nucleus or by the presence of distinct nuclei associated with axonemes (Figure 1d). We counted the number of activated male or female gametocytes of the WT and *gep(‐)*cl1 with either a ruptured PVM and/or RBCM (Figure 1e). This revealed striking differences between the two parasite lines. About 90% of the WT male gametocytes had a breached PVM, and 70.5% were fully released from the host cell, while 50% of the *gep*(‐) males had vesiculated PVM and in only 5.5% the RBCM ruptured. In females, distinguished by their smaller nucleus, we detected similar results whereby only 4% of the *gep(‐)* females were fully released, while 38% had a ruptured PVM compared with the WT in which 91% had a ruptured PVM and 69% breached RBCM (Figure 1d,e).

### 3.3 GEP is expressed in male and female gametocytes and partly localizes to OBs

To determine the expression pattern and subcellular localization of GEP protein, a specific serum was produced against a recombinant peptide expressed in *E. coli*.

**Figure 2:**
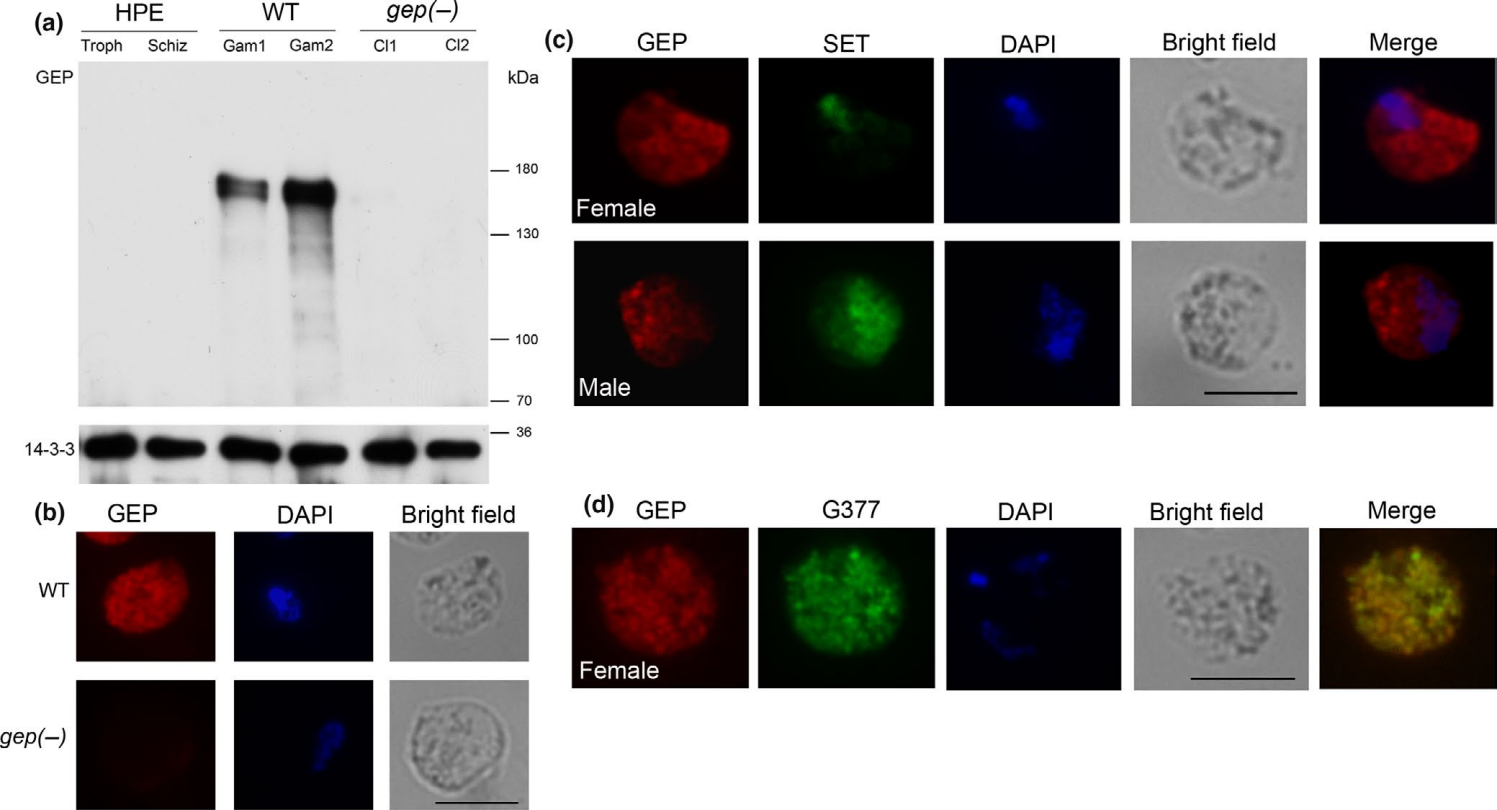
(a) Western blot analysis on purified HPE parasites, WT gametocytes (2 independent preparations), and *gep(‐)* gametocytes (clones 1 and 2). GEP serum recognizes a specific band at 133 kDa in the WT gametocyte samples, while no signal was detected both in asexual parasites (HPE) and in gametocytes of the *gep(‐)* cloned lines. Samples were normalized using anti‐Pb14‐3‐3 detected in both asexual and sexual stages (Lalle et al., 2011). (b) Immunofluorescence assay using GEP‐specific serum. A signal is detected in WT gametocytes but not in mutant parasites. Scale bar 5 μm. (c) Double IFA on WT gametocytes using immune sera against GEP serum and the nuclear protein SET, highly abundant in male gametocytes. Scale bar 5 μm. (d) Double IFA using anti‐GEP and anti‐G377 as a marker of OBs. Scale bars 5 μm

In a Western blot assay (Figure 2a), a band at the predicted size of 133 kDa was detected in WT gametocytes samples from two independent preparations but no signal was detected in the two *gep(‐)* clones neither in blood samples of the *P. berghei* line HPE which does not produce gametocytes (Janse, Ramesar, Berg, & Mons, 1992), indicating that GEP is expressed specifically in sexual stages. 14‐3‐3 was used as a control to normalize sample amount (Lalle et al., 2011) .

In IFA, the specific serum detected GEP in a punctated pattern in WT gametocytes while no signal was seen in *gep(‐)* clones (Figure 2b). GEP serum labels both female and male gametocytes (Figure 2c) identified by costaining with anti‐SET serum, highly abundant in the nuclei of male gametocytes (Pace et al., 2006). Double IFA using anti‐GEP and anti‐G377 immune sera showed that GEP localizes partly to OBs (Figure 2d).

### 3.4 *Gep(‐)* gametocytes are delayed in OB release upon activation of gametogenesis

**Figure 3:**
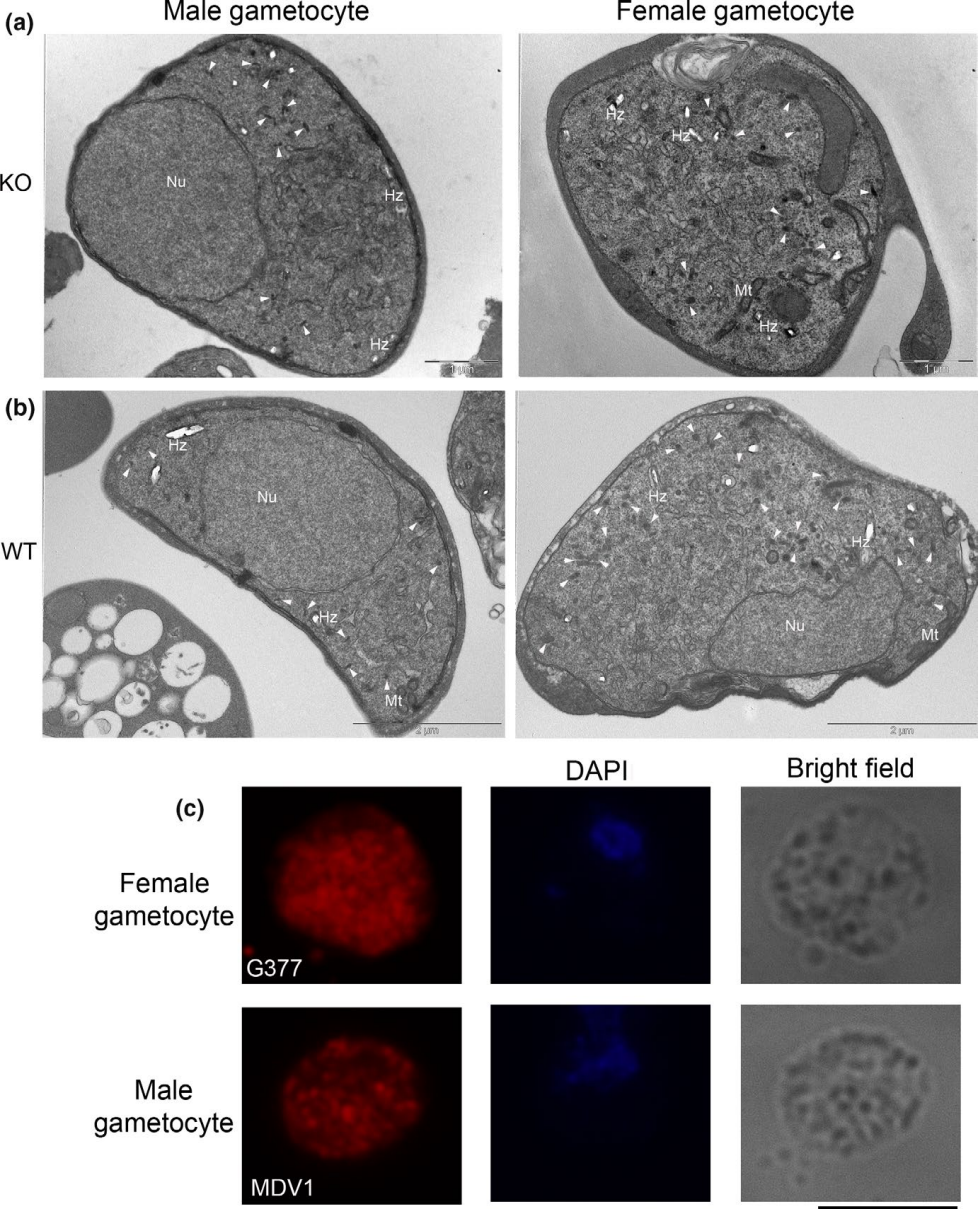
(a) Ultrastructural analysis of male and female gametocytes of *gep(‐)* parasites. The cells display the typical appearance of gametocytes. In males, an eccentric and very large nucleus (Nu), a lot of typical club‐shaped male osmiophilic bodies (Mobs) and of hemozoin crystals (Hz), both randomly distributed in the cytoplasm, and the inner membrane. In females pronounced rough endoplasmic reticulum (ER), hemozoin granules and abundant oval‐shaped osmiophilic bodies (OBs) scattered in the cytoplasm. Scale bar 1 μm. (b) WT ultrastructure section of male and female gametocyte. Nu: Nucleus; arrowhead: MOBs in male and OBs in female gams; Hz: hemozoin; (c) IFA on *gep(‐)* parasites: a female gametocyte (upper panel) stained with the immune serum against the OB marker G377 and a male gametocyte stained with the immune serum against the OB marker MDV. Scale bar 5 μm

**Figure 4:**
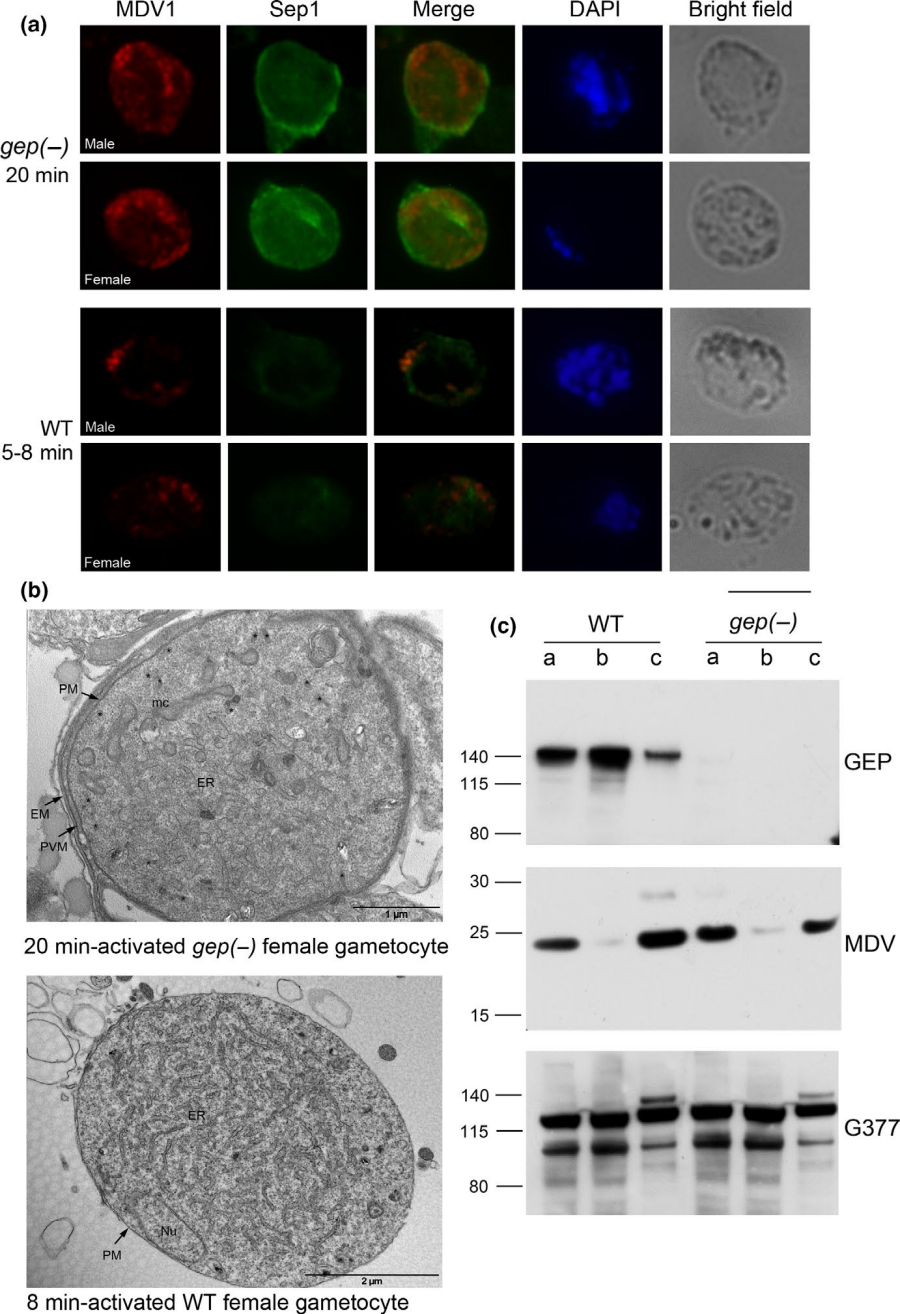
(a) Double IFA of activated WT and *gep(‐)* gametes labeled with the PVM marker SEP1 and the OB marker MDV1. In the WT, OBs discharged their content 5–8 min postactivation with the concomitant disruption of the PVM. In the *gep(‐)* cl1 gametocytes, an intact PVM and OBs still inside the cells are visible. Scale bar 5 μm. (b) Ultrastructure of *gep(‐)* female gametocyte activated 20 min (upper panel); the PVM and erythrocyte membrane (EM) are intact and OBs (asterisks) are present in the cytoplasm. WT female gametocyte activated 8 min (bottom panel) fully egressed. (c) Western blot analysis using GEP, MDV1, and G377 sera on samples prepared from purified WT and *gep(‐)* gametocytes (lane a), gametes (lane b), and exflagellation supernatant (lane c). The samples correspond to 10^7^ gametocytes purified from synchronous infections. Gametes and supernatant were from samples activated for an extended time (30 min). GEP is partly secreted during gamete activation as demonstrated in the upper panel of the WB. At this time point, OBs protein content is normally secreted in *gep(‐)* parasites, as revealed in the blots probed with MDV1 serum. G377 is partly retained in the gametes and partly secreted

Ultrastructural analysis of *gep(‐)* gametocytes indicated no evident morphological alteration of organelles, including male and female OBs (Figure 3a,b). IFA staining confirmed the correct localization of the OB‐resident proteins G377 and MDV1 to punctate structures in gametocyte cytoplasm (Figure 3c). However, in the activated *gep(‐)* gametocytes, double IFA with SEP1 and MDV1 (Figure 4a) showed that OBs move to the cell periphery but do not efficiently discharge their content, as suggested by the presence of an intact PVM 20 min postactivation (Figure 4a, upper panel). In contrast, in WT parasites, MDV1 is almost completely released in 5–8 min, with the concomitant disruption of the PVM (Figure 4a, lower panel). This was also confirmed by ultrastructural analysis of activated WT and mutant female gametocytes (Figure 4b), showing the presence of OBs in the mutant at 20 min after activation.

To verify whether the lack of GEP either abrogates or causes inefficient OB release, we extended the activation time of both WT and *gep(‐)* gametocytes to 30–35 min and evaluated the egress efficiency by Western blot analysis comparing the amount of the OB‐resident proteins MDV1 and G377 released in the exflagellation medium. As shown in Figure 4c, the amount of released OB markers in knockout parasites is comparable with that of WT, suggesting that the egress process is delayed in *gep(‐)* parasites but not abrogated. In the activated gametocytes of the WT, only a small amount of GEP was released in the exflagellation medium, while the large majority of the protein was still detected inside the activated gametocytes (Figure 4c). This suggests that the role of GEP in the egress process may not require an efficient secretion of the protein. It is conceivable that GEP takes part in events preceding OB discharge, for example, promoting membrane fusions. This may explain the delay in OB release, observed in *gep* null parasites.

### 3.5 *Gep(‐)* male gametes form normal axonemes, but flagella are not motile

**Figure 5:**
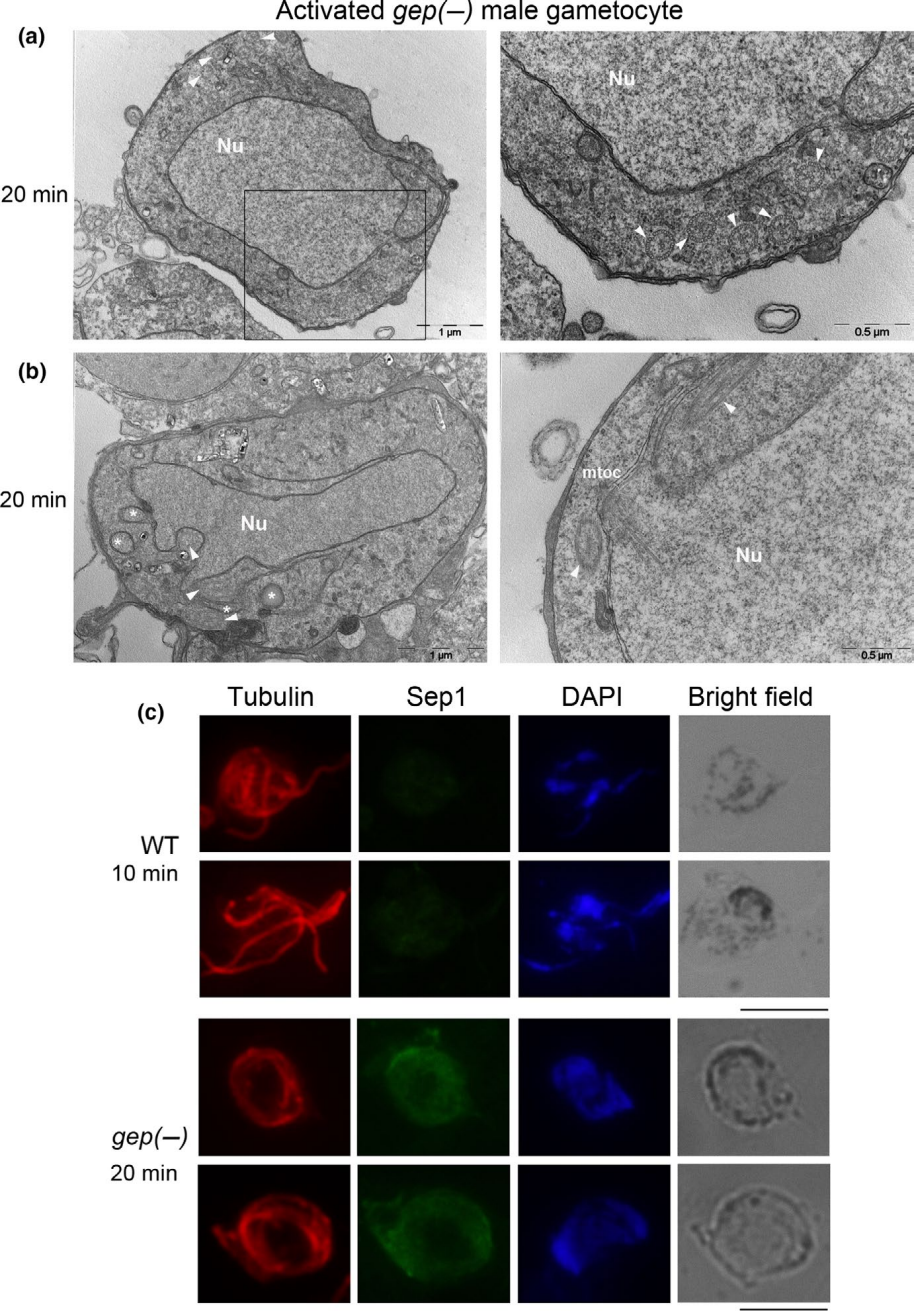
(a) Morphological EM section of an activated *gep(‐)* male gametocyte 20 min after induction of gametogenesis. The RBCM is intact as well as the PVM. In the male cell, normally structured axonemes (arrowhead) are seen. (b) Morphological EM section of activated *gep(‐)* male gametocyte. In this section, a center of the microtubular organization is showed (MTOC) as well as nuclear lobes (asterisks). (c) IFA on WT 10 and 20 min activated and *gep(‐)* activated gametocytes at 20 min after induction stained with anti‐tubulin and SEP1 sera. In red, tubulin is detected on the flagella of WT and *gep(‐)* gametes. In the mutant samples, PVM is detected (labeled with SEP1) and tubulin is visible in a pattern consistent with nonmotile axonemes. Enlarged nuclei are also visible suggesting

As shown above, in Figure 1c, we did not see a single exflagellation event in the activated *gep(‐)* gametocytes even though a fraction of male gametes emerged from the host erythrocyte (Figure 1e). By microscopic inspection (Videos S1 and S2), we observed that flagella, although visible, were not motile in the *gep(‐)* mutant while moving actively in WT gametes. This could be due to a defect in the structural organization of flagella or timing of assembly of their components. To distinguish between these possibilities, we investigated the formation of the axonemes in *gep(‐)cl1* activated male gametocytes by electron microscopy. The ultrastructural analysis confirmed that the majority of observed male gametocytes were still inside the PVM 20 min postactivation (an exemplar image is shown in Figure 5a). In the cytoplasm of activated males, axonemes were visible with the typical ring of nine outer microtubule doublets (Figure 5a, right panel). However, we did not observe a concomitant nuclear division but an enlarged multilobated nucleus and only in a few cases microtubule organization centers (Figure 5b).

The same preparations of activated WT and *gep(‐)*gametocytes were also examined in parallel by IFA using anti‐tubulin antibodies that stain male gamete flagella (Figure 5c). In the WT, gamete formation was almost completed 10 min postactivation. In *gep(‐)* parasites*,* male gametocytes were still enclosed in the membranes 20 min postactivation. Distinct nuclei were not visible while axonemes were fully formed consistent with the EM data. Overall, these data suggest that in *gep(‐)* mutant, axoneme formation and nuclear divisions are not coupled.

### 3.6 Genetic crosses reveal a functional defect in both genders of *gep(‐)*

**Figure 6:**
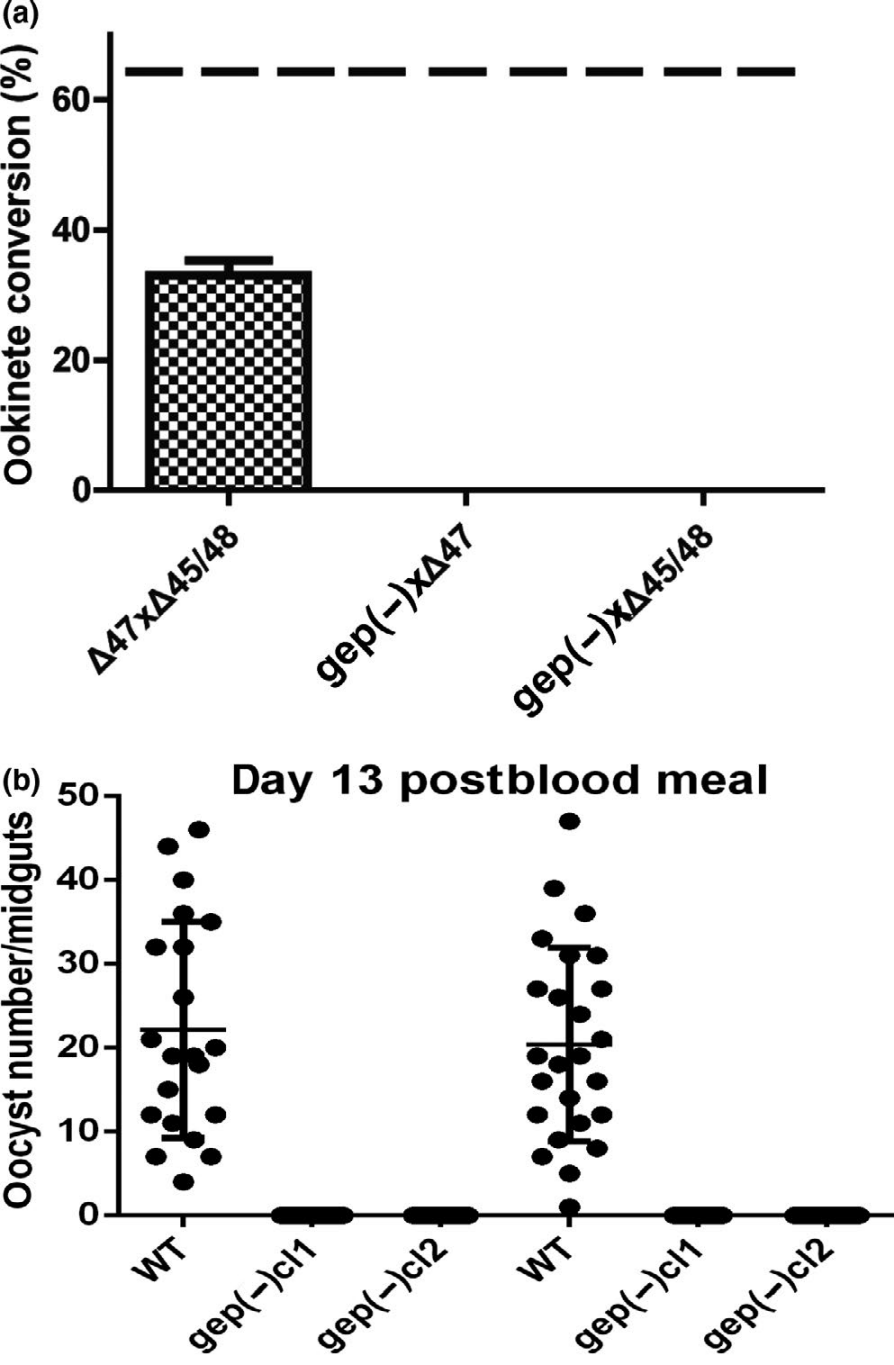
(a) Ookinete conversion from genetic crosses of *gep(‐)* parasites and parasite lines producing sterile male (Δ45/48) and female (Δ47) gametocytes. As a control, Δ45/48 was also crossed to Δ47. Dotted line: average WT ookinete conversion. b) Oocyst formation of WT and *gep(‐)*. In mosquitoes infected with *gep(‐)* parasites, no oocysts were detected in dissected mosquito midguts 13 days after blood feeding

Our experiments indicated that in *gep(‐)* transgenic line, both male and female gametocytes were affected in egress and that male gametocytes did not form motile flagella suggesting an additional role of GEP, not limited to the breakdown of cell membranes during gametogenesis. To determine whether the absence of GEP affects ookinete formation, we carried out genetic crosses by mixing infected blood of the *gep(‐)*cl1 and parasite lines forming either only fertile males (Δ47) or fertile females (Δ48/45) (van Dijk et al., 2010). After incubation overnight, we counted the number of ookinetes formed from the female gametocytes. Results of crosses showed in Figure 6 indicated that both male and female gametes of the *gep(‐)* line were unable to produce ookinetes in three independent experiments, while a cross of Δ47 and Δ48/45 done as a control produced fully mature ookinetes.

To check the in vivo phenotype of *gep(‐)* parasites, *An. gambiae* mosquitoes were fed on mice infected with WT or *gep(‐)* cloned lines. As expected, no oocysts were detected in 2 independent experiments using the two *gep(‐)* clones, while in both experiments, WT parasites were infective to mosquitoes (Figure 6b).

## 4 DISCUSSION

In this study, we identified the protein GEP (PBANKA_1115200) as a novel factor required for the successful gametogenesis of malaria parasites. GEP is a well‐conserved protein within the *Plasmodium* genus, but without any similarity to known proteins of other organisms pointing to a highly specific function in malaria parasites. The protein was observed in the cytoplasm of gametocytes partly colocalized with an osmiophilic body (OB) protein. Our functional analysis revealed that mutants lacking *gep* had a pleiotropic phenotype with defects in gametogenesis resulting in the inability of both genders to establish mosquito infections. Both male and female gametocytes had a severe delay in egress from the red blood cell. At 20 min postactivation, the PVM was still intact in about half of the cells. A more dramatic effect was seen in RBCM rupture with only a few mutant parasites being released from the host cell. Furthermore, during male gametogenesis, no motile flagella were formed, although axonemes were assembled. The ultrastructural analysis confirmed this finding and also revealed that nuclear division did not take place. Genetic crosses confirmed that GEP was essential for the fertility of both genders.

Gametogenesis is a multi‐step process, with many aspects unique to the malaria parasite. In both genders, the coordinate action of several *Plasmodium*‐specific proteins is required for egress of mature gametes from the host RBC. In *P. berghei,* OB‐resident proteins MDV1 (Ponzi et al., 2009) and GEST (Talman et al., 2011) are necessary for membrane rupture and in mutants lacking either of these proteins mature gametes of both genders remain trapped inside the host cell. OBs move to the cell membrane after activation, and cargo proteins are secreted in the PV lumen 5–8 min upon gametocyte activation. While *gep(*‐) parasites are also severely affected in egress from the host cell, in contrast to MDV and GEST, the protein is mostly retained inside the cytoplasm of the gametocytes. However, we cannot exclude a minor portion of the protein being secreted due to the lack of a suitable marker to investigate this aspect. We observed that OBs also in our mutants moved to the cell membrane, but the release of cargo was delayed though not abolished. This suggests that GEP has a role upstream of OB discharge. Interestingly, a function in membrane fusion and secretion has been shown for the OB‐resident small solute transporter PAT (Kehrer, Singer, et al., 2016). Similar to GEP, the phenotype of mutant parasites lacking PAT is a severe reduction in the release of gametes of both genders.

Male gametogenesis is a complex process with three mitotic divisions taking place concomitantly with axoneme assembly. After cytokinesis, axonemes are activated and eight flagellar gametes, each with a haploid nucleus, exit the cell. In the *gep(‐),* mutant axonemes were formed but not activated and cytokinesis did not take place. Similar phenotypes have been observed previously. Functional studies on the Anaphase Promoting Complex/Cyclosome (APC/C) revealed a role in cell cycle control during male gametogenesis; a conditional mutant was severely affected in chromosome condensation and cytokinesis (Wall et al., 2018). A similar phenotype was also observed in *P. berghei* mutants lacking the CDC20 homolog (Guttery et al., 2012) and the MAP2 kinase (Tewari et al., 2005). The possibility that GEP together with these three proteins is part of a common pathway is an interesting hypothesis.

GEP has also been identified as interacting with P granule proteins ALBA4 (Muñoz et al., 2017), DOZI (Mair et al., 2006), and the Sm‐like factor CITH (homolog of worm CAR‐I and fly Trailer Hitch, Mair et al., 2010) the latter two which are required in females for proper development of the zygote (Mair et al., 2010).

P granules store and protect mRNA and function in post‐transcriptional repression of transcripts previously produced in the parasite. Noteworthy is that the phenotype of the mutant lacking GEP is different from mutants lacking DOZI, CITH, and ALBA4. A mutant lacking ALBA4 showed an increase in male exflagellations but had no apparent function in female gametocytes (Muñoz et al., 2017) while DOZI and CITH mutants were blocked in zygote development, through misregulation of maternal transcripts. A possible function of GEP in transcript regulation remains to be investigated.

How membrane rupture, flagellar beating, and cytokinesis are coordinated during male gametogenesis is still largely unknown. GEP may represent a valuable tool to further investigate this aspect in the field of gamete egress.

## CONFLICT OF INTEREST

None declared.

## AUTHOR CONTRIBUTION

Maria Andreadaki: Data curation (equal); Validation (equal). Tomasino Pace: Data curation (equal); Methodology (equal); Validation (equal). Felicia Grasso: Methodology (supporting). Inga Sidén‐Kiamos: Conceptualization (supporting); Writing‐original draft (supporting). Stefania Mochi: Methodology (supporting). Leonardo Picci: Methodology (supporting). Lucia Bertuccini: Methodology (supporting). Marta Ponzi: Conceptualization (equal); Data curation (equal); Funding acquisition (equal); Writing‐original draft (supporting). Chiara Currà: Conceptualization (lead); Data curation (equal); Formal analysis (equal); Funding acquisition (supporting); Investigation (equal); Project administration (equal); Supervision (equal); Writing‐original draft (lead); Writing‐review & editing (lead).

## ETHICS STATEMENT

All work was carried out in full conformity with Greek regulations and laws on animal experiments. In Greece, these issues are covered by the Presidential Decree (160/91) and law (2015/92) which implement the directive 86/609/EEC from the European Union and the European Convention for the protection of vertebrate animals used for experimental and other scientific purposes and the new legislation Presidential Decree 56/2013. The experiments were carried out in a certified animal facility license (EL91‐BIOexp‐02), and the protocol has been approved by the FORTH Ethics Committee and by the Prefecture of Crete (license number # 93491, 30/04/2018).

## Data Availability

All data are provided in full in the results section of this paper except the DNA sequence for PBANKA_1115200 available at PLASMODB: https://plasmodb.org/plasmo/app/record/gene/PBANKA_1115200. The supplementary movie files, Video S1 and Video S2, are available in Zenodo at https://doi.org/10.5281/zenodo.3751384

## APPENDIX 1

**Table A1:** List of primers

Primer name	Sequence
L‐f (111520‐KO‐L‐f)	gaccccgcggagtaaaatttcctgacctgtc
L‐r (111520‐KO‐L‐r)	gaccgaattcggtatccatatttctttaagtg
R‐f (111520‐KO‐R‐f)	gaccctcgaggactactctcagtcgttacac
R‐r (111520‐KO‐R‐r)	gaccggtacctacacactttcacacgtgcc
A (111520‐L‐ext)	gattatgctatgatgtgtgt
B (111520‐R‐ext)	tgtatgtgtgcctatcattgc
C (111520‐L‐int)	ggggtttgaattctggagcg
D (111520‐R‐int)	gtctaatcttcttgttgtcg
E (mcherry‐bk)	atgatggccatgttatcctc
*F* (5’pbdhfr‐bk)	gcatggggaaagagtggctt
Rel‐10‐for	gaccggatcccccaaaatgaatgaagatcgcg
Rel‐10‐rev	gaccgcggccgcgtcgacttcgttactagtcattgac

**Table A2:** WT and *gep(‐)* gametocytemia scored in Giemsa

	% male gametocytes	% female gametocytes
WT	1.1 ± 0.8	1.2 ± 0.8
*gep(‐)* clone 1	0.9 ± 0.7	1.1 ± 0.8
WT	2.1 ± 1.6	1.9 ± 0.7
*gep(‐)* clone 2	1.6 ± 0.7	0.9 ± 0.2

**Note:** The study presented here refers to a protein GEP (gamete egress protein). After acceptance of this article, we became aware that a publication Nat Commun 11, 1764 (2020). https://doi.org/10.1038/s41467-020-15479-3 describes an independent study and a different protein named GEP1 (gametogenesis essential protein 1).
